# High content screening of patient-derived cell lines highlights the potential of non-standard chemotherapeutic agents for the treatment of glioblastoma

**DOI:** 10.1371/journal.pone.0193694

**Published:** 2018-03-02

**Authors:** Kenny Kwok-Hei Yu, Jessica T. Taylor, Omar N. Pathmanaban, Amir Saam Youshani, Deniz Beyit, Joanna Dutko-Gwozdz, Roderick Benson, Gareth Griffiths, Ian Peers, Peter Cueppens, Brian A. Telfer, Kaye J. Williams, Catherine McBain, Ian D. Kamaly-Asl, Brian W. Bigger

**Affiliations:** 1 Brain Tumour Research Group, Stem Cell and Neurotherapies Laboratory, Division of Cell Matrix Biology & Regenerative Medicine, University of Manchester, Manchester, United Kingdom; 2 Manchester Centre for Clinical Neurosciences, Salford Royal Hospital, Manchester Academic Health Sciences Centre, Salford, United Kingdom; 3 Imagen Therapeutics, Manchester, United Kingdom; 4 Inferstats Consulting, Alderley Park, Biohub, Cheshire, United Kingdom; 5 Division of Pharmacy & Optometry, School of Biology, Medicine and Health, University of Manchester, Manchester, United Kingdom; 6 Department of Clinical Oncology, The Christie NHS FT, Manchester, United Kingdom; 7 Children’s Brain Tumour Research Network (CBTRN), Royal Manchester Children’s Hospital, Manchester, United Kingdom; 8 Department of Neurosurgery, Royal Manchester Children’s Hospital, Manchester Academic Health Sciences Centre, Manchester, United Kingdom; Northwestren University, UNITED STATES

## Abstract

**Background:**

Glioblastoma (GBM) is the most common primary brain malignancy in adults, yet survival outcomes remain poor. First line treatment is well established, however disease invariably recurs and improving prognosis is challenging. With the aim of personalizing therapy at recurrence, we have established a high content screening (HCS) platform to analyze the sensitivity profile of seven patient-derived cancer stem cell lines to 83 FDA-approved chemotherapy drugs, with and without irradiation.

**Methods:**

Seven cancer stem cell lines were derived from patients with GBM and, along with the established cell line U87-MG, each patient-derived line was cultured in tandem in serum-free conditions as adherent monolayers and three-dimensional neurospheres. Chemotherapeutics were screened at multiple concentrations and cells double-stained to observe their effect on both cell death and proliferation. Sensitivity was classified using high-throughput algorithmic image analysis.

**Results:**

Cell line specific drug responses were observed across the seven patient-derived cell lines. Few agents were seen to have radio-sensitizing effects, yet some drug classes showed a marked difference in efficacy between monolayers and neurospheres. *In vivo* validation of six drugs suggested that cell death readout in a three-dimensional culture scenario is a more physiologically relevant screening model and could be used effectively to assess the chemosensitivity of patient-derived GBM lines.

**Conclusion:**

The study puts forward a number of non-standard chemotherapeutics that could be useful in the treatment of recurrent GBM, namely mitoxantrone, bortezomib and actinomycin D, whilst demonstrating the potential of HCS to be used for personalized treatment based on the chemosensitivity profile of patient tumor cells.

## Introduction

Glioblastoma (GBM) is the most common and biologically aggressive primary brain malignancy in adults, with a median survival of 14.2 months. Current treatment comprises of maximal surgical resection followed by radiotherapy with concomitant temozolomide (TMZ)[1]. However, despite treatment, long term survival in GBM is rare, with the recurrent tumor usually resistant to treatment[2]. There is currently little consensus on the optimal treatment regimen for recurrent GBM (rGBM). Procarbazine, lomustine and vincristine (PCV) combination therapy[3], irinotecan[4] and carmustine impregnated wafers[5] are all used in clinical practice, yet in the absence of a strong evidence base, rGBM therapy is largely palliative.

Glioma stem-like cells (GSCs), a multipotent, self-renewing subpopulation of cells within the tumor microenvironment, are believed to be responsible for disease recurrence. These cells are resistant to both radiotherapy and chemotherapy[6] and are able to recapitulate the molecular and phenotypic characteristics of primary GBM in neurosphere culture[7], thus are promising targets for screening therapeutic options *in vitro*.

High content screening (HCS) allows phenotypic changes in cell morphology to be rapidly assessed and analyzed and the resulting multi-parametric readout allows for the build-up of a more comprehensive picture of cell fate when exposed to drug treatments. In the short-term, combining a drug repositioning strategy with HCS could prove a useful tool for matching the best currently available chemotherapy regimen to a particular patient; however, a standardized and physiologically relevant *in vitro* model must be proven in order for it to be useful in the clinic.

Previous small-molecule screens using GBM cell lines have relied on adherent culture and immortalized cell lines to achieve the standardization needed for such large-scale assays[8, 9]. Through the use of patient-derived GSCs and serum-free culture, the phenotypic and genetic diversity of the parental tumor can be maintained more accurately[10]. However, adherent culture can create abnormal cell morphology and cells often show increased sensitivity to small molecules compared to the same cells grown on matrices or other three-dimensional culture systems[11, 12]. Previous studies have attempted to answer the question of whether allowing patient-derived cells to form neurospheres, or inducing adherence using laminin results in the same pattern of inhibitor sensitivity[13, 14]. Yet to date, a thorough comparison of chemosensitivity across both culture methods in this format has not been performed.

We have therefore established a 384-well high content screen that enabled us to assess the chemosensitivity profiles of patient-derived cell lines in both monolayer and neurosphere culture against a panel of 83 chemotherapeutics, with and without irradiation. Our study puts forward several non-standard chemotherapeutics that show efficacy *in vivo* and have the potential to be rapidly entered into clinical trials.

## Materials and methods

### Patient characteristics

Patients with pre-operative imaging characteristics in keeping with glioblastoma were identified through the neuro-oncology multi-disciplinary meeting at Salford Royal NHS Foundation Trust. Informed consent was obtained from patients for tissue use, storage and derivation of cell lines. All work was performed with NHS research ethics committee approval (Ref. 68/H1006/38 and 08/H1006/37). Patient demographic features are summarized in Table 1.

**Table 1:** Patient demographics, clinical features and cell line designations.

Cell Line Designation	Location	Histology^a^^,[^^15^^]^	Primary Treatment	Treatment at Recurrence	Overall Survival (Months)
**GS1**	Right fronto-temporal	Gliosarcoma	Surgical resection	N/A	4.3
**TS1**	Left frontal & thalamic	Gliomatosis cerebri	Biopsy only	N/A	1.3
**TS6**	Right frontal	GBM	Surgical resection + TMZ +Irradiation	PCV	9.2
**TS9**	Left frontal	Recurrent GBM	Surgical resection + TMZ +Irradiation	Surgical resection +PCV	14.3
**TS17**	Right occipital	GBM	Surgical resection	N/A	0.9
**TS18**	Left parietal	GBM	Surgical resection	N/A	5.6
**TS20**	Left frontal	GBM	Surgical resection + TMZ +Irradiation	N/A	8.4

^a^Histological diagnoses were made prior to the WHO 2016 re-classification. Gliomatosis cerebri is now referred to as a rare diffuse-pattern glioblastoma and gliosarcoma has been incorporated into the umbrella term IDH-wildtype glioblastoma.

### Chemicals

Eighty-three chemotherapeutic drugs (S1 Fig) were purchased from Selleck Chemicals, USA and reconstituted in DMSO at a stock solution of 10mM. *In vivo* drugs were purchased from Sigma Aldrich, USA and dissolved in DMSO at a stock solution of 80mM and diluted further in sterile saline for administration as indicated. Cell viability experiments were performed via the fluorescent dyes Hoechst 33342 (Sigma Aldrich, USA) and DRAQ7 (Biostatus, UK) and fixed with 1% paraformaldehyde solution (Sigma Aldrich, USA).

### Patient cell line derivation and cell culture

Patient cell line derivation was in accordance with Pollard et al.[16]. Dissociated tumor cells were cultured in Neurobasal medium (NBM) supplemented with N2, B27, EGF, and FGF-2 (20 ng/ml). Laminin was added to the media at 25ng/ml to allow for cell adherence. Cells were maintained in a standard tissue culture incubator (37°C; 5% CO2) and media and growth factors replenished regularly until cells reached 90% confluence. Neurosphere forming ability was tested by passaging in supplemented NBM in the absence of laminin. Spheroids typically formed within 24hrs. The U87-MG (U87) immortalized cell line was maintained in DMEM supplemented with FCS, L-glutamine and penicillin streptomycin in adherent culture. U87 neurospheres were formed by replacing complete DMEM with supplemented NBM.

### Immunocytochemistry

Neurospheres were cultured over 48 hours then fixed in 4% PFA; 0.1% Triton-X. Neurospheres were then dehydrated through a methanol gradient and incubated in 100% methanol overnight, then rehydrated via the same methanol gradient. Neurospheres were then blocked in 3% fish skin gelatin; 0.1% Triton-X for four hours. Primary antibodies were used at the following dilutions: Nestin 1:1000 (Abcam) and Sox-2 1:250 (Abcam). Secondary staining was performed using Alexa-Fluor 488 and 594 secondary antibodies (Invitrogen), followed by nuclear counterstaining with DAPI (300mM).

Images were collected sequentially on a Leica TCS SP5 AOBS upright confocal using a 20x / 0.50 Plan Fluotar objective and 3x confocal zoom. When acquiring 3D optical stacks the confocal software was used to determine the optimal number of Z sections. Only the maximum intensity projections of these 3D stacks are shown in the results.

### Immunohistochemistry

Immunohistochemistry was performed on free floating sections treated with 10% goat serum for 2 hours at room temperature, and then stained with primary antibodies for GFP 1:1000 (Abcam), vimentin 1:200 (Abcam) and human nuclear antibody 1:2000 (Abcam). Primary antibodies were detected using the appropriate secondary antibodies.

### GFP labelling of patient derived cell lines

Patient cell line TS18 was stably transfected with a lentivirus containing an eGFP cassette driven by the SFFV promoter[17]. Transduced cells were expanded in culture, then sorted to select for intensity of eGFP expression over yield using a BD FACS Aria flow cytometer (BD Biosciences, UK).

### High content screen

#### Cell preparation

Cells were grown to confluence then disaggregated and diluted to 2.5x10^4^ cells / ml in supplemented NBM +/- laminin. Cells were then dispensed using a Multi-drop liquid dispenser into black 384-well clear, flat bottom plates to achieve a seeding density of 1 x 10^3^ cells in 40μl per well. Neurosphere plates were then incubated for 7 days at 37°C in a humidified incubator with 5% CO_2_, whilst laminin-treated plates were incubated for 48 hr. under the same conditions. U87 cells were seeded in complete DMEM for adherent culture and NBM (- laminin) for neurosphere culture.

#### Drug treatment

Eighty-three chemotherapeutic agents were dosed on to cells according to a 5-point log dosing strategy (0.001, 0.01, 0.1, 1 and 10μM) using a liquid handling robot (Hamilton Star) and drugs dosed in quadruplicate in a 2x2 pattern. Each plate included 14 positive (staurosporine; 0.1μM) and 14 vehicle (DMSO; 0.1%) controls. Half the plates were then exposed to a single dose of irradiation in a biological irradiator (5 Gy; X-Rad 320, Precision X-ray Inc., USA). Plates were then incubated for 96 hours at 37°C in a standard tissue culture incubator before incubation with the fluorescent dyes Hoechst 33342 (1:500) and DRAQ7 (1:1000) for 30 minutes at 37°C. Cells were then fixed in 1% paraformaldehyde (PFA) solution.

#### High content image acquisition and analysis

Image acquisition was performed using the Cellomics ArrayScan VTI HCS Reader (Hamamatsu ORCA®-ER camera with x5 objective lens) and images analyzed using the Cellomics package software (Cellomics, USA). The ArrayScan software algorithm was used to perform compartmental analysis and objects below a certain size excluded from analysis. Regions of Interest (ROI) were identified using the software. DRAQ7fluorescence and ROI were overlaid to determine the amount of DRAQ7 associated with each neurosphere or cell. Hoechst dye incorporation was counted by the software and used count cells and neurospheres.

### *In vivo* studies

NOD-SCID Gamma Null (NSG) mice between 8 and 12 weeks of age were used in this study. Mice were housed in individually ventilated cages with ad libitum access to food and water, and were kept in a 12-hour light/dark cycle. Female mice were used in this study and housed in groups of 2–5. All procedures were ethically approved by the University of Manchester Ethical Review Process under UK Home Office regulations and project license PPL 40/3658.

#### Tumorigenicity studies

TS1-GFP, GS1, TS9 and TS18-GFP cells (2.5x10^5^) were stereotactically injected into the right striatum of NSG (n = 4 per cell line). Mice were sacrificed when they became symptomatic or suffered significant weight loss (>20% weight loss over 24 hr). After trans-cardial perfusion, brains were harvested, fixed in 4% PFA and cryoprotected with 20% sucrose. Coronal sections were taken with a Hyrax M25 rotary microtome (Carl Zeiss, Germany) and prepared on glass slides for immunohistochemical staining.

#### Drug validation studies

Female NSG mice, aged ≥8 weeks, were implanted with subcutaneous xenografts using U87-MG cancer cells (5 x 10^6^). Palpable tumors were established approximately 10 days after cells were injected. Tumors reached 200mm^3^ approximately 18 days post injection, at which point drug treatment was initiated.

Drugs were administered at sub-maximum tolerated doses. Mitoxantrone, 12mg/kg, single dose i.v. bortezomib, 1mg/kg, i.p. at day 1 and day 4; actinomycin D, 0.5mg/kg, single dose i.v.; paclitaxel 3.79 mg/kg i.p. days 1–4; doxorubicin 4mg/kg, single dose i.v. and temozolomide 50mg/kg i.p. days 1–5. A DMSO vehicle group was included (5% DMSO, i.v) as well as a non-treated control group. Dose, drug delivery method and scheduling of each drug was in accordance with accepted published values to maintain sub-maximal tolerated doses in mice[18–24].

To monitor tumor growth, xenografts were measured (major and minor axis) every two days using calipers, and tumor volume was calculated using the equation:

$$\frac{{(a}^{2}\;x\;b)}{2}$$

Where a = width and b = length of the tumor. Mice were culled seven days post-treatment and tumors excised. Final tumor volumes were measured and plotted against controls.

### Data analysis

For monolayer assays cell death was defined as the percentage of cells with DRAQ7 positivity. Defined by setting an upper “circ spot” intensity using the Cellomics compartmental analysis algorithm (Thermofisher, USA). Spheroid count was defined as the number of filtered particles identified in neurosphere culture through compartmental analysis. The three-dimensional nature of the neurospheres was taken into account by normalizing the total DRAQ7 intensity against spheroid cross-sectional area. Due to variation and non-normal distribution, the values were incremented by 1 and log transformed. Therefore, cell death in neurosphere assays was defined as:

$$Cell\;Death=Log(\frac{DRAQ7\;Intensity}{Spheroid\;Cross\;Sectional\;Area}+1)$$

For response space analysis, the average log cell death and spheroid count was calculated across all concentrations for each compound. The y-axis represents the difference in cell death between drug and negative control. On the x-axis, differences were expressed as fold change over DMSO as they represented relative rather than absolute differences over DMSO. 95% confidence limits were calculated using Spotfire (Tibco Software, USA) and data collated and visualized using Microsoft Excel (Microsoft, USA). pEC50 values were extrapolated from unambiguous dose-response curves generated from DRAQ7 raw data. Dose response curves, heat maps and Bland-Altman plots were generated using Prism (v.7.0, Graphpad Software, USA).

## Results

### Derivation of patient GBM cell lines

**Fig 1:**
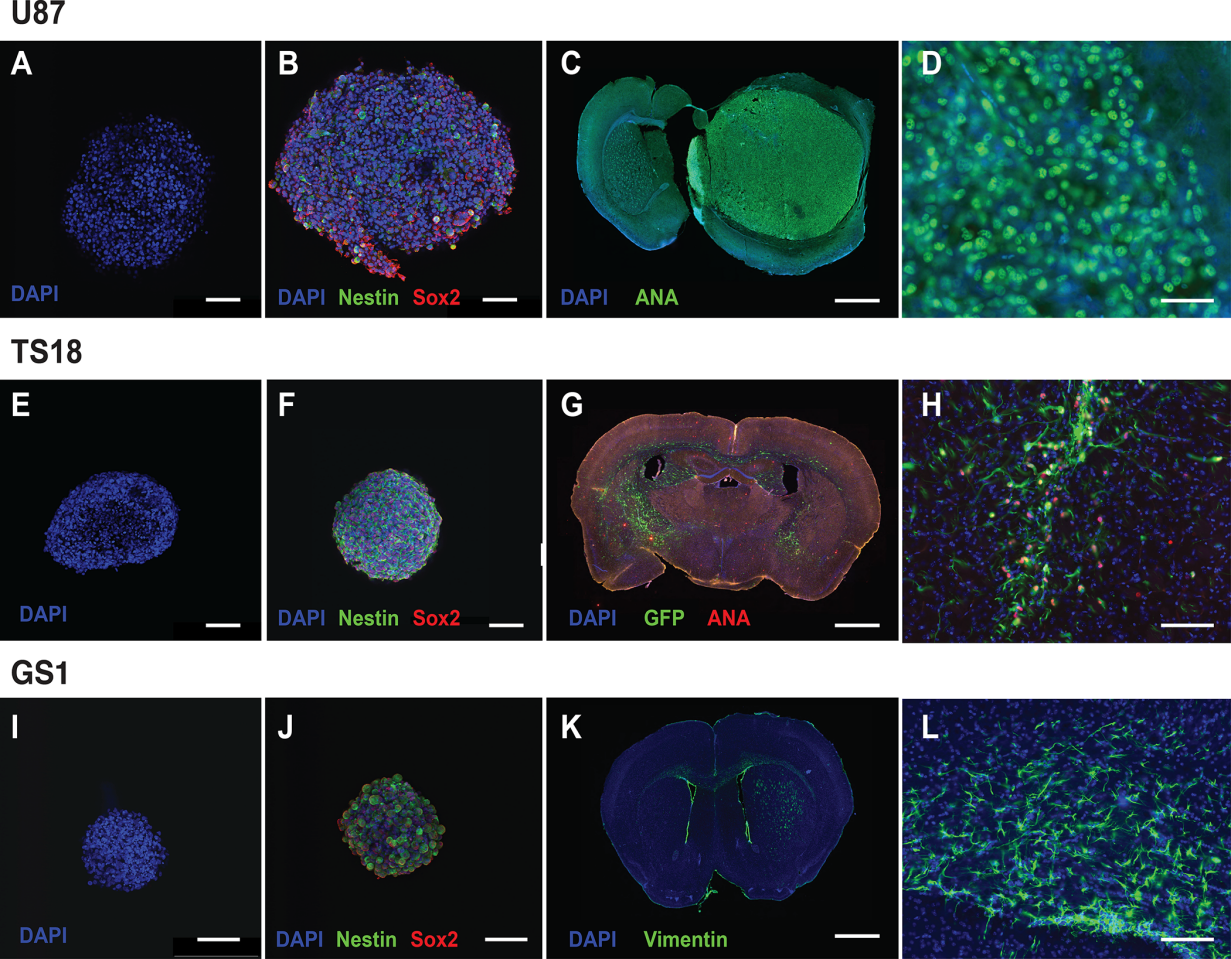
Cell line characterization demonstrated stem-cell characteristics and tumorigenicity *in vivo*. (A-B) U87 neurosphere culture demonstrating Nestin (green) and Sox2 (red) staining in serum-free culture (s.b. = 100μm). (C) U87 orthotopic implantation model showing large well-defined masses within the brain parenchyma, showing staining for anti-human nuclear antigen (green) (s.b. = 1000μm). (D) High power field (x40) of well-demarcated tumour edge showing anti-human nuclear antigen staining of tumour cells (s.b. = 50μm). (E-F) TS18 in serum free neurosphere culture showing uniform Nestin and Sox2 staining (s.b. = 100μm). (G) Orthotopic implantation of TS18-GFP cells with combined GFP (green) and anti-human nuclear antigen (red) staining to show diffuse infiltration of cells both within the parenchyma and across the corpus callosum into the opposite hemisphere (s.b. = 1000μm). (H) High power field (x20) showing area of diffuse infiltration and co-expression of GFP and anti-human nuclear antigen in implanted cells (s.b. = 100μm). (I-J) GS1 neurosphere culture showing uniform Nestin and Sox2 staining in serum free culture. (K) Orthotopic implantation of GS1 cells into the striatum with anti-vimentin staining. Vimentin staining is characteristic of gliosarcoma and is not found in the parenchyma, and was used to trace GS1 cells within the parenchyma (s.b. = 1000μm). (L) High power field (x20) showing diffusely infiltrating vimentin positive cells (green) within the corpus callosum (s.b. = 100μm).

Cell lines were successfully generated from primary tumor tissue from seven patients with GBM. All patient-derived lines were examples of primary GBM, with the exception of TS9 which is a recurrent line. Each line was characterized for stem-like properties. Patient-derived cell lines were cultured in serum-free conditions with and without laminin,and formed neurosphere-like aggregates when laminin was not present in the media (Fig 1A, 1E and 1I). U87 monolayer culture was achieved through culturing in complete medium with serum. Neurosphere cultures all showed positive immunocytochemical staining for Nestin and Sox2 (Fig 1B, 1F and 1J), demonstrating that derived lines expressed putative stem-cell markers. Orthotopic implantation of all patient lines used in the screen was also conducted in NSG mice, to demonstrate the cell line’s ability to initiate tumor growth *in vivo* (Fig 1C, 1G and 1K).

### *In vitro* drug screening

To both identify drugs that are cytotoxic for glioma stem-like cells and to assess the most physiologically relevant screening model, drugs were screened in two distinct assay models; neurosphere cultures or laminin-induced monolayers. Our platform also enabled us to assess whether any of our chemotherapeutics were radio-sensitive through exposing half the assay plates to a single dose of irradiation (5Gy). After 96 hours incubation with drug, with or without irradiation, cells were fixed and stained with DRAQ7 and Hoechst prior to imaging and data collection.

**Fig 2:**
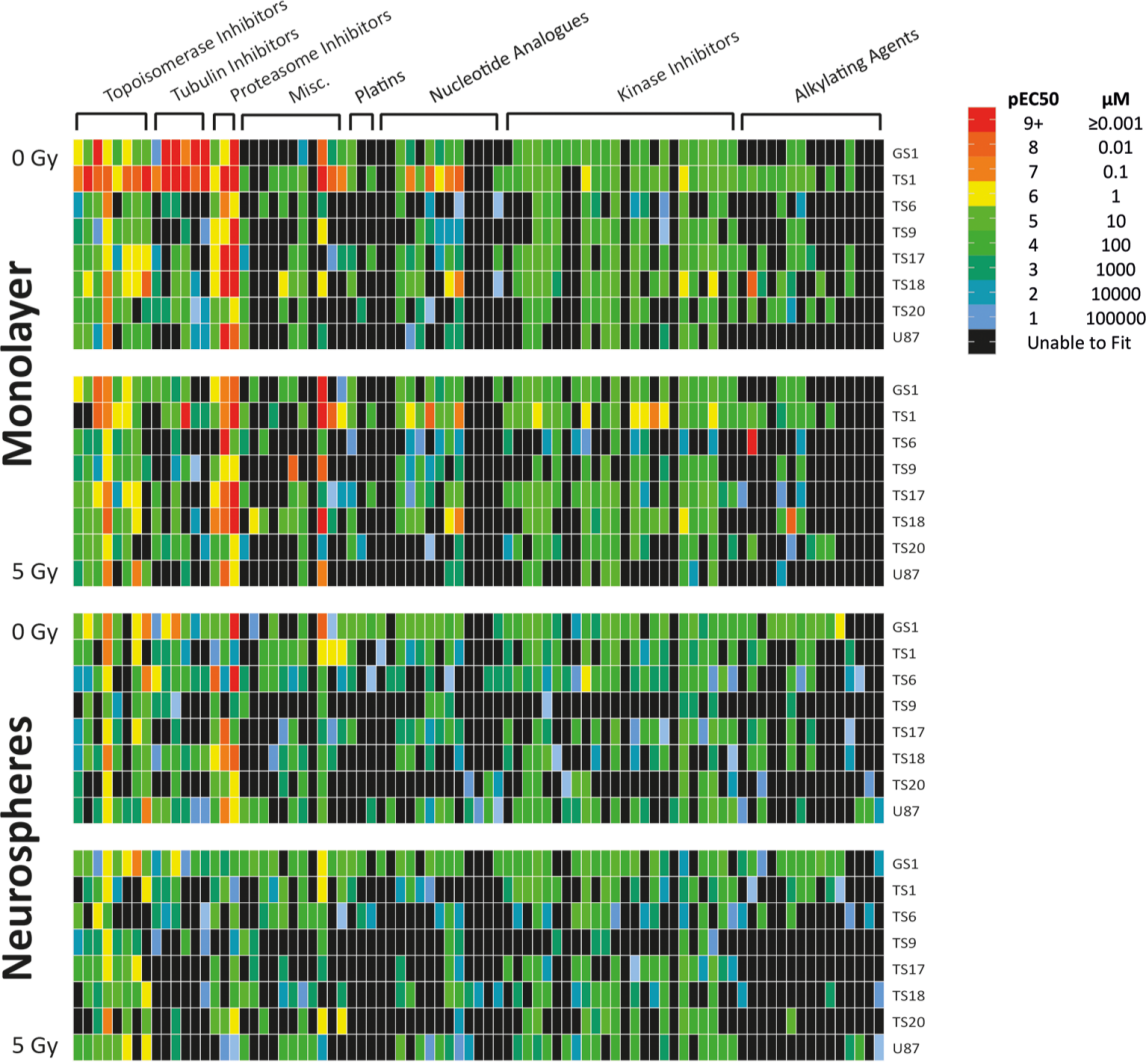
pEC50 values highlighted general resistance of GBM cell lines and cell line specific responses to chemotherapeutics and irradiation. Heat Map was populated using best-fit pEC50 values generated from dose-response curves, then color mapped categorically according to the legend. Each heat map color and corresponding number (1,2,3 etc.), corresponds to a log change in concentration of drug (100,000 μM, 10,000 μM, 1000 μM etc.). Red values of a pEC50 of 9 or above would indicate an EC50 of 1nm or below, indicating high efficacy, whereas any values categorized green-blue or below would likely be poor drug candidates as they correspond to EC50 values above 10μM. Any data Prism was unable to fit was colored black—this was typically due to an inability to generate an EC50 with that drug candidate because of inefficiency at high concentrations.

pEC50 values were generated via percentage cell death or log cell, based on DRAQ7 positivity. DRAQ7 does not cross the membrane of viable cells and thus is a marker of cell membrane permeabilisation, apoptosis, necrosis and dead cells. A heat map was populated using the best-fit pEC50 values to compare the four assay models (Fig 2). A full list of screened drugs and pEC50 values can be seen in S1 Fig. Across all four assay models, pEC50s were generated for an average of 55% of chemotherapeutics in each cell line, reflecting the inherent resistance of GSCs. However, a total of 37% of drugs produced pEC50s below 100μM across all eight lines.

TS1 was the most sensitive cell line in adherent culture, with 78.3% of drugs with a pEC50 corresponding to less than 100 μM without irradiation, and 60% after a single 5Gy dose. TS9, our one recurrent line, was resistant to most compounds tested in all assay formats, with only 21% of drugs generating a pEC50 below 100 μM over all four assays. This low percentage most likely reflects the fact that the patient the cells were derived from had both first line irradiation plus Temozolomide (TMZ), followed by PCV combination therapy at recurrence.

The more commonly used GBM chemotherapeutic compounds, including temozolomide with radiotherapy, showed varying success across cell lines in our screen. None of our assays indicated >10% efficacy at 10μM in TS9 or U87, therefore no pEC50 data could be generated. This lack of response in the U87 cell line is consistent with previous studies [14, 25], whereas in TS9 this is most likely due to therapy-induced resistance. Radio-sensitization of cells to specific drugs was rare, although a general increase in the efficacy of kinase inhibitors was observed, notably dasatinib efficacy significantly increased in both TS9 and TS17 post-irradiation.

Vincristine demonstrated good efficacy across all patient-derived lines, with the exception of TS9. GS1 was particularly sensitive, with pEC50s corresponding to low nanomolar efficiency in both monolayer and neurosphere assays. Lomustine and procarbazine were less efficacious alone. Irinotecan, another common second line option, was much more successful, with the majority of drugs demonstrating cytotoxicity in the low micromolar or nanomolar range.

In addition to irinotecan, other topoisomerase inhibitors were cytotoxic across the majority of cell lines, with the most consistently efficacious drug being mitoxantrone. pEC50s were obtained in each cell line, ranging between 2.7nM and 87.5μM. Indeed, along with tubulin and proteasome inhibitors, all sub-nanomolar pEC50 values were found in these classes, along with 56% of drugs with an pEC50 corresponding to less than 100 μM.

### Method comparison of assay models

**Fig 3:**
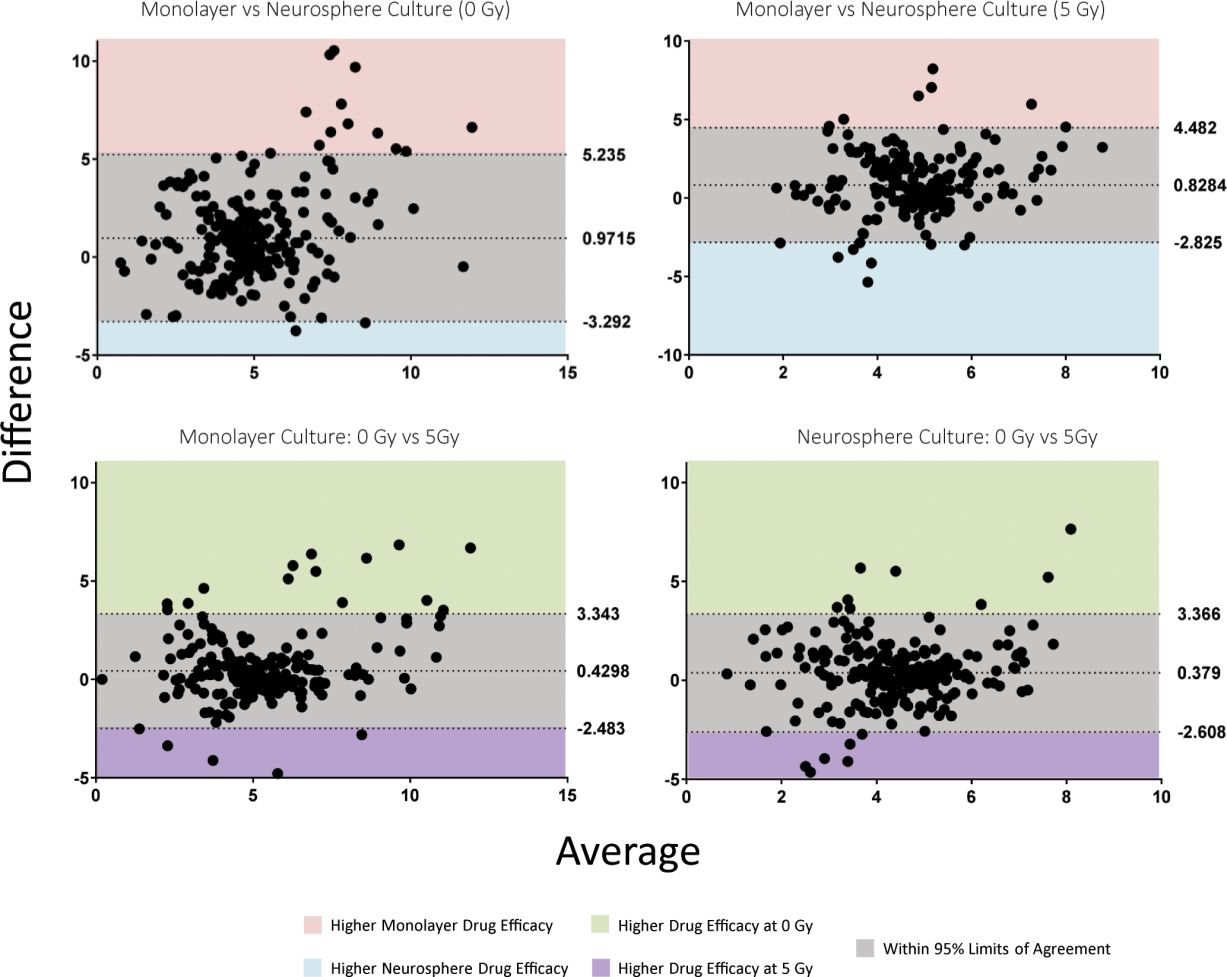
Bland-Altman method comparison of assay models demonstrated no overall bias for potency, whilst highlighting specific drugs to take forward for validation. All pEC50s were stacked by patient-derived line. Mean difference and lower and upper limits of agreements (95% CI) are indicated by dotted lines. Values outside these limits can be considered real biological differences.

Bland-Altman method comparison plots were used to assess the degree of agreement between the four assays and demonstrated no overall bias for potency between neurosphere and monolayer culture, nor between irradiated and non-irradiated plates (Fig 3). Mean differences in pEC50 were in the range of 0.3–0.5 and between 9.6 and 19.2% of compounds were within 95% limits of agreement.

### *In vivo* validation

For *in vivo* studies, pEC50’s for the U87 cell line were ranked and drugs were chosen that either showed high efficacy or ranked markedly different in one assay type over another. In the U87 cell line, bortezomib and mitoxantrone had a high pEC50 value, corresponding to high drug cytotoxicity, in both culture models. Doxorubicin and paclitaxel were more efficacious in the monolayer assay, whilst actinomycin D was ineffectual in the monolayer assay, yet had a high pEC50 in the neurosphere assay. This panel of drugs was additionally validated in an alternative assay read out (S1 File) for activity in the U87 cell in both culture formats (S2 Fig), with similar pEC50 discrepancies seen between the two methods.

**Fig 4:**
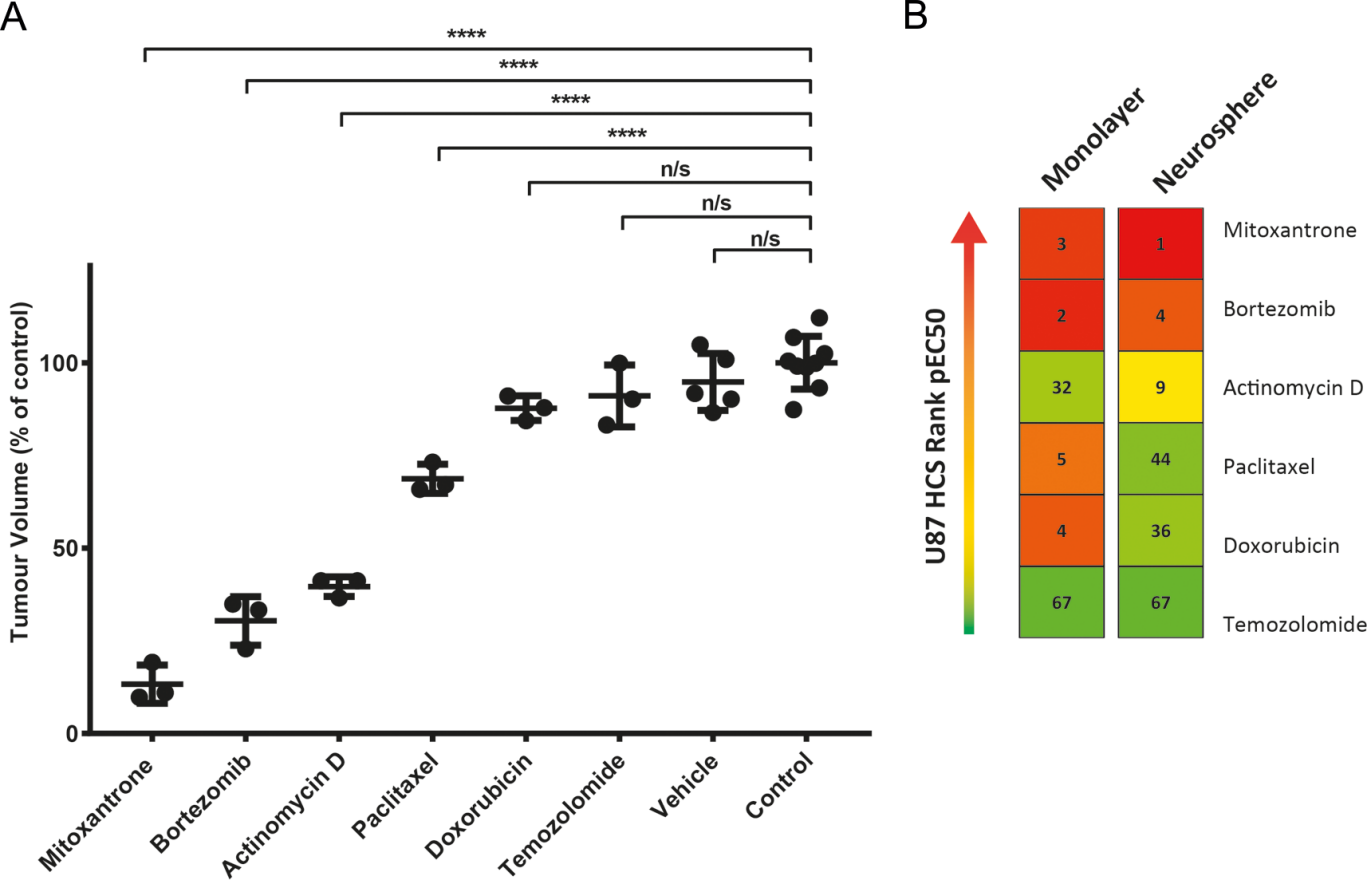
*In vivo* assessment of drug activity demonstrated strong correlation with neurosphere *in vitro* results. (A) NSG mice were dosed at sub-maximal tolerated doses with reference to accepted published data^18-24^. Tumors were excised seven days post-treatment then measured ex-vivo for accuracy. Results are expressed as the percentage volume compared to the control group (*p < 0.05, **p < 0.01, ***p < 0.001 and **** p < 0.0001). (B) Rank order of drugs based on pEC50 values for the U87 cell line are shown here to facilitate comparison. pEC50s were generated for 66 drugs in one, or both, of the U87 screening models, thus rankings are from 1 to 67, with 1 having the highest pEC50 and therefore the highest cytotoxicity. Mitoxantrone, bortezomib and actinomycin D all ranked in the top 10 in the neurosphere assay, which appears to correlate with the *in vivo* efficacy seen in these three drugs. Paclitaxel and doxorubicin demonstrated low efficacy *in vivo*, yet ranked highly in the monolayer assay.

*In vivo* results (Fig 4A) demonstrate that treatment with vehicle, TMZ and doxorubicin at sub-MTD had almost no effect on the inhibition of tumor growth *in vivo*, consistent with the low ranking seen in neurosphere assays. Mitoxantrone, actinomycin D and bortezomib all had a strong inhibitory effect on tumor growth, with mitoxantrone proving the most effective. Although paclitaxel showed evidence of high efficacy in monolayer assays, this effect was not as marked *in vivo*, with an inhibitory effect of ~30%. Overall, where compared with the rank order seen in Fig 4B, the neurosphere HCS assay appeared to reflect *in vivo* effect more accurately than the adherent assay in this model system.

### Chemosensitivity profiling of patient-derived cell lines

**Fig 5:**
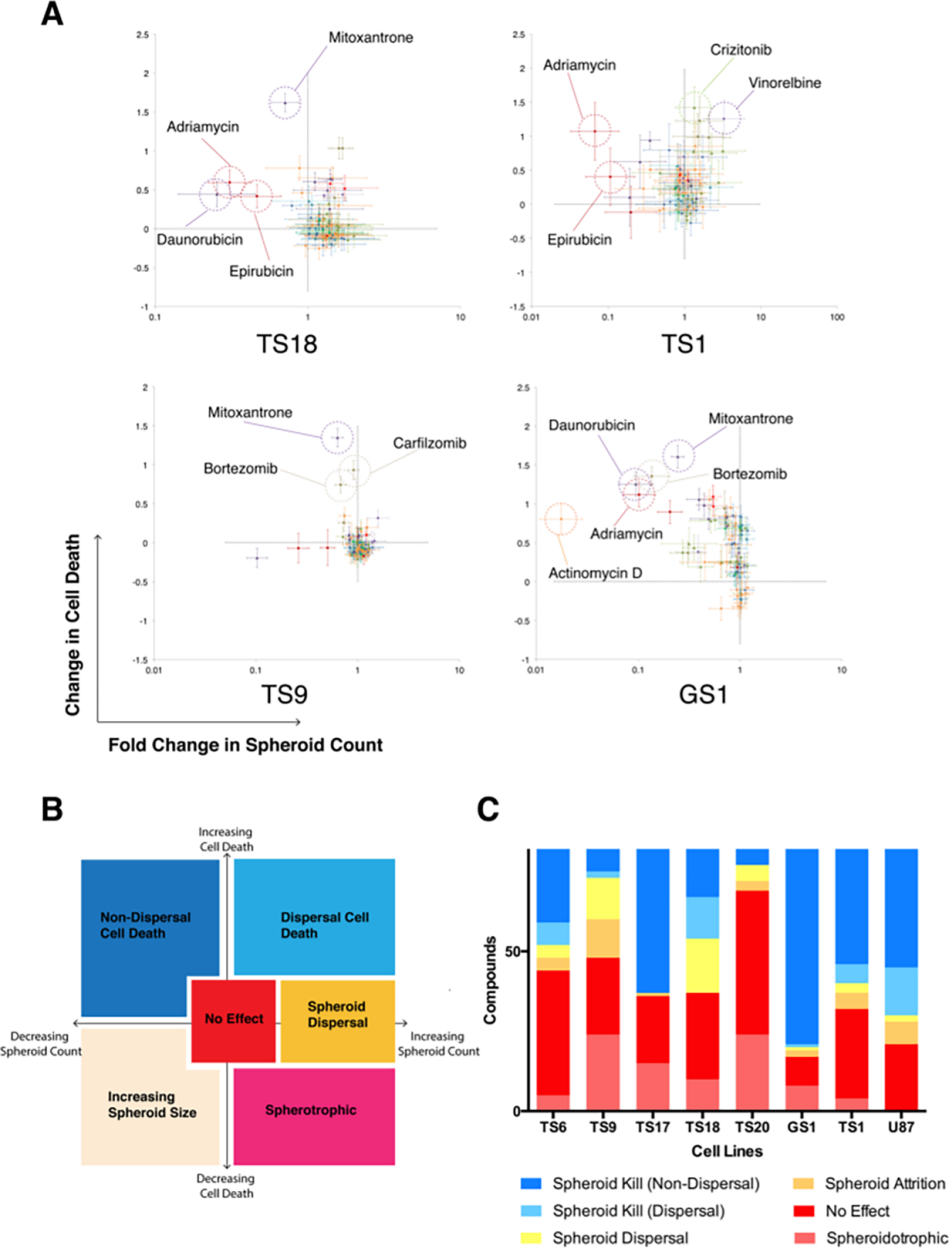
Response space analysis identifies highly effective drugs and patient cell–line resistance. (A) Responses for TS1, TS18, TS9 and GS1 (0Gy) are depicted. Response is plotted onto a response space for each drug by the change in cell death (as measured by DRAQ7 intensity/spheroid cross sectional area), and fold change in neurosphere count. Highly responsive drugs are highlighted in dashed circles, with negative controls sitting at the intersection of the dotted lines (0, 1). (B) Sub -classification of drug response in neurosphere culture. Blue shaded areas represent responsive drugs, and yellow/orange/red areas represent resistance. (C) Summarized neurosphere response sub-classifications. The combined height of the yellow/orange/red columns provides an indication of the overall chemotherapeutic resistance of the cell line.

Although cell death is a useful parameter for stratifying drugs for *in vivo* validation, HCS is a multi-parametric technique, and captures additional data including morphology and spheroid count. Through plotting cell death against spheroid count we developed a response space plot for each cell line in neurosphere culture to stratify drug response (Fig 5).

Response space analysis allows immediate identification of highly effective drugs, as these compounds are significantly separated from negative controls, with more sensitive lines having a larger number of drugs in the upper left quadrant, whereas drugs tend to cluster around DMSO controls in the more resistant cell lines. Prior treatment effects appear to be reflected in the response space (Fig 5B). TS9, derived from rGBM, is characterized by marked non-response in the majority of drugs. The few drugs that do show response are mitoxantrone, bortezomib, and carfilzomib respectively, with effect appearing to be sustained with irradiation.

## Discussion

In this study, we demonstrate the feasibility of using high content screening techniques to generate chemosensitivity data on patient-derived cell lines within a reasonable timeframe of 4 weeks. Although chemosensitivity assays have been performed in GBM previously[13, 14], our screen was performed in multiple patient-derived GSC lines. With each line cultured in both monolayer and neurosphere culture and exposed to all screened drugs and graded irradiation. Our platform’s two primary outputs were cell death (as measured by overall DRAQ7 intensity), and cell/spheroid count. Since we did not know *a priori* which of these output parameters would be more representative of tumoricidal effect, we chose to perform analysis on both outputs in a Response Space. This approach not only highlighted drugs which showed typical cell killing effect—cytotoxicity with concomitant reduction in spheroid count, but also highlighted specific drugs (e.g. axitinib, ponatinib) with atypical response patterns, such as an increase in cell death with paradoxical increase in spheroid count–a phenomenon we termed ‘spheroid dispersal’. Although the significance of this observation was not clear, it was interesting to note that drugs within the same class (tyrosine kinase inhibitors) tended to cluster together within the Response Space, which also provided indirect evidence that the assay system was capturing a true biological effect. Finally, the Response Space also allows us to immediately identify highly effective drugs, as well as highly resistant cell lines, as exemplified by the rGBM cell line TS9.

Radio-resistance of GSCs is well documented; nonetheless, a general increase in efficacy of kinase inhibitors, post-irradiation, was noted. Dasatinib inhibits the PI3K/Akt/GSK3 pathway downstream of BCR/ABL. Dasatinib efficacy was significantly increased in two of our patient lines, corresponding with pre-clinical data demonstrating radio-sensitization of GSCs through Akt inactivation [26]. Thus, through optimization of dosing schedules and incubation periods, this model has the potential to investigate radio-resistance in GSCs with targeted kinase-inhibitor libraries.

Direct comparison of cell lines in monolayer and neurosphere culture demonstrated that the two formats are analogous. However, there were isolated differences with certain drugs. Our *in vivo* drug panel was chosen to investigate whether these specific differences were physiologically relevant. Using the U87 cell line, we demonstrate that when pEC50 values are ranked, only the results from our neurosphere assay corresponded to *in vivo* results. An increased sensitivity to chemotherapeutics in adherent, monolayer cell cultures has been demonstrated before [27–29]. It is possible that the proliferation rate of cancer cells grown in 3D cultures more accurately reflects *in vivo* tumor growth[30]. Thus, our study suggests that the chemosensitivity of cancer cells is culture specific for GBM, which may influence the interpretation of chemosensitivity results in standard monolayer formats currently used in high throughput drug discovery.

Mitoxantrone was the most efficacious chemotherapeutic across all cell lines, demonstrating extremely high levels of cytotoxicity, even at very low concentrations. Mitoxantrone is a type II topoisomerase inhibitor which induces expression of p53 resulting in cellular apoptosis. Mitoxantrone proved to reduce tumor size by an average of 86% compared to controls at sub-maximal tolerated doses in NSG mice. Unfortunately, clinical trials in high grade gliomas in the 1980s using systemic mitoxantrone chemotherapy failed to show similar results[31].

Cisternino et al.[32] have shown limited penetration of mitoxantrone into the CNS. There is however promise that local delivery of mitoxantrone can be effective, with administration of mitoxantrone concomitantly with PCV and irradiation in rGBM showing an increase in long-term survival[33]. In addition, pre-clinical studies using strategies such as siRNA to specifically inhibit drug efflux transporters have shown that drug delivery to orthotopic brain tumors can be dramatically increased[34, 35]. Additionally, irinotecan is known to have only limited distribution across the blood brain barrier, yet has had modest success as a monotherapy in GBM[36], and whilst studies show the tissue to blood ratio of TMZ is remarkably low in the brain, this does not appear to reduce its efficacy or utility as standard therapy[37].

Alongside mitoxantrone, several other drugs identified in our study were also ABC-transporter substrates, hence the reason we chose to primarily validate the efficacy of our drugs in a sub-cutaneous *in vivo* model. Few studies have investigated the efficacy of actinomycin D in high grade glioma, although a prior study has indicated that it is effective in glioma cells through the activation of caspase-2 and -3 mediated apoptosis[38]. Actinomycin D is used to treat a variety of childhood cancers, including neuroblastoma and has been previously trialed in pediatric atypical teratoid/rhabdoid tumors[39]. Furthermore, low dose actinomycin D has recently been shown to restore p53 function in RELA-positive ependymoma[40].

Conversely, bortezomib has been shown to be effective against glioblastoma cells in numerous pre-clinical models and its efficacy has been attributed to various mechanisms[41, 42]. However, the promise of bortezomib *in vitro* has yet to be reflected in clinical trials, with two phase II trials combining bortezomib with tamoxifen[43] and verinostat[44] proving ineffectual. A recent phase II study has also demonstrated that combination bortezomib and TMZ was ineffective in rGBM despite promising pre-clinical data[45]. Measurable concentrations of bortezomib were however found in the tumor tissue post-treatment, strengthening our view that ABC-transporter substrates can penetrate the BBB in GBM.

Our HCS platform also demonstrates benefit in the field of rare variants of glioblastoma. We generated chemosensitivity data for a diffuse-pattern glioblastoma cell line (TS1) and a glioblastoma line, diagnosed by a neuropathologist, to have a sarcomatous histopathological appearance (GS1). Robust clinical trial data for these variants is often unavailable, as the scarcity of patients makes trial accrual difficult. HCS therefore could potentially provide a rational basis for therapy selection in these cases.

## Conclusion

This proof-of-principle screen has identified and validated *in vivo* a number of non-standard chemotherapeutics that have the potential to be effective in the treatment of primary and rGBM, namely mitoxantrone, bortezomib and actinomycin-D. Whilst trials involving systemic administration of mitoxantrone and bortezomib have been unsuccessful, the possibility of directly delivering agents within the tumoral cavity using techniques such as convection enhanced delivery and implantable polymers render the ability to cross the BBB a secondary concern in the selection of effective drugs[46]. The limited data available on the use of actinomycin-D in adult cancers make it an interesting drug to explore in further detail for the treatment of rGBM.

This work represents the initial steps in acquiring, handling and processing chemosensitivity data on an HCS platform. *In vivo* data suggests that a three-dimensional assay platform gives the most physiologically relevant data; therefore, future work will concentrate on optimizing this model to produce a reproducible and rapid screening model with the potential to generate clinically actionable information.

## Supporting information

S1 Fig pEC50 values highlighted general resistance of GBM cell lines and cell line specific responses to chemotherapeutics and irradiation. Heat Map was populated using best-fit pEC50 values generated from dose-response curves, then color mapped categorically according to the legend. Each heat map color and corresponding number (1,2,3 etc.), corresponds to a log change in concentration of drug (100,000 μM, 10,000 μM, 1000 μM etc.). Red values of a pEC50 of 9 or above would indicate an EC50 of 1nm or below, indicating high efficacy, whereas any values categorized green-blue or below would likely be poor drug candidates as they correspond to EC50 values above 10μM. Any data Prism was unable to fit was colored black—this was typically due to an inability to generate an EC50 with that drug candidate because of inefficiency at high concentrations.
(TIF)

S2 Fig U87 chemosensitivity measured via the presto blue assay. Dose response curves for five non-standard chemotherapeutics identified in the primary HCS plus the standard GBM therapeutic, temozolomide. Data are presented as the mean cell viability compared to mean vehicle control of two replicate assays (six wells per dose) ± standard error of the mean.
(TIF)

S3 Fig Neurosphere response space analysis for each cell line. Each drug response is separated into change in cell death on the Y-axis (as measured by DRAQ7® intensity/spheroid area), and fold change in spheroid count in the X-axis. Each cell line is treated with drugs only (left), and with irradiation (right). Drug classes are shown below the chart. Gy = Gray.
(TIF)

S1 Table U87 EC50 values measured via the presto blue assay. The antilog of logEC50s extrapolated from dose response curves were used to populate the table, along with 95% confidence intervals for each EC50. Curves were fitted and EC50 values extrapolated using GraphPad Prism (v7.0).
(TIF)

S1 File Supporting information. Additional methods and description of supporting information figures and tables.
(DOCX)
