# Supplementary figures and images for: High content screening of patient-derived cell lines highlights the potential of non-standard chemotherapeutic agents for the treatment of glioblastoma
Source: PLoS One. 2018 Mar 2;13(3):e0193694. doi: 10.1371/journal.pone.0193694 (PMC5834163; doi:10.1371/journal.pone.0193694)

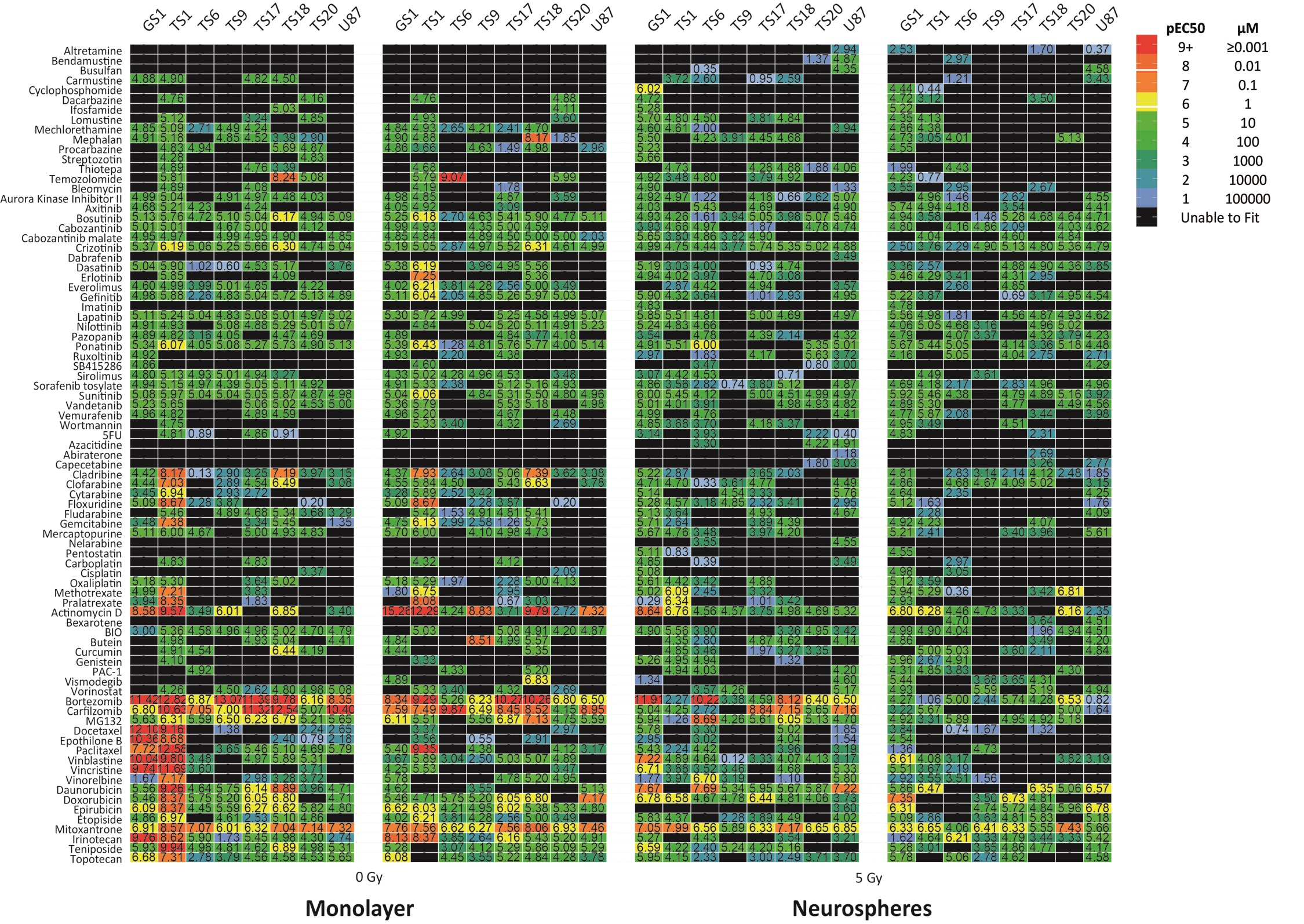

Supplement: S1 Fig — Heat Map was populated using best-fit pEC50 values generated from dose-response curves, then color mapped categorically according to the legend. Each heat map color and corresponding number (1,2,3 etc.), corresponds to a log change in concentration of drug (100,000 μM, 10,000 μM, 1000 μM etc.). Red values of a pEC50 of 9 or above would indicate an EC50 of 1nm or below, indicating high efficacy, whereas any values categorized green-blue or below would likely be poor drug candidates as they correspond to EC50 values above 10μM. Any data Prism was unable to fit was colored black—this was typically due to an inability to generate an EC50 with that drug candidate because of inefficiency at high concentrations. (TIF) [file pone.0193694.s001.tif]

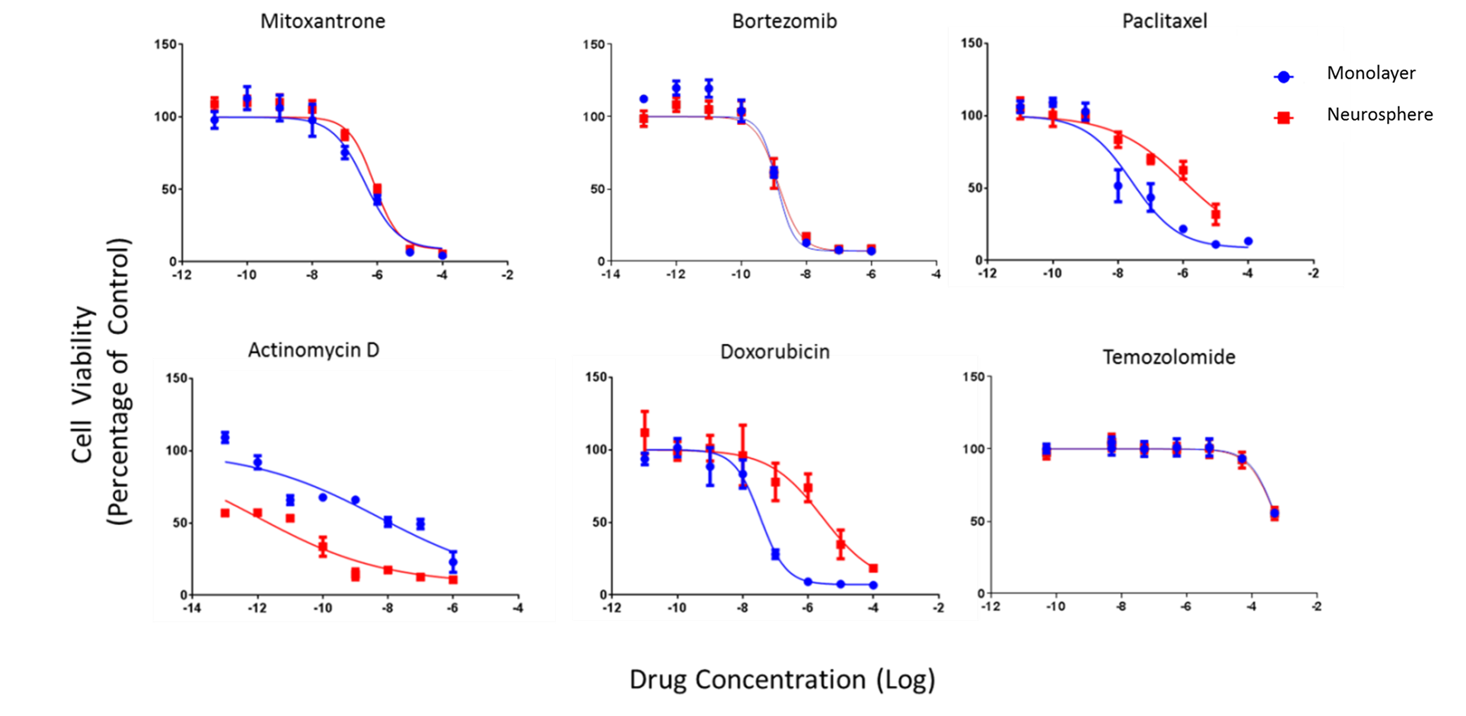

Supplement: S2 Fig — Dose response curves for five non-standard chemotherapeutics identified in the primary HCS plus the standard GBM therapeutic, temozolomide. Data are presented as the mean cell viability compared to mean vehicle control of two replicate assays (six wells per dose) ± standard error of the mean. (TIF) [file pone.0193694.s002.tif]

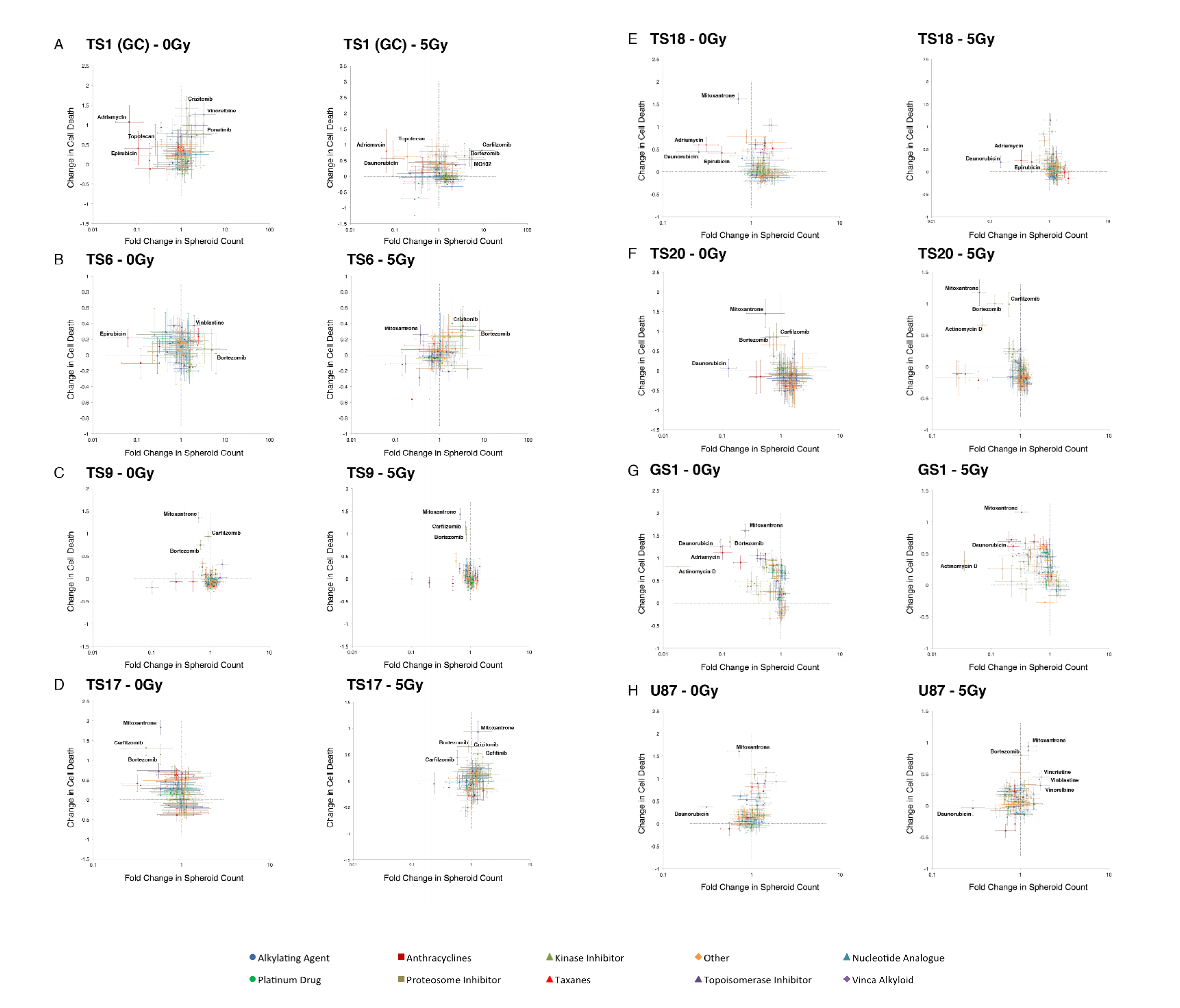

Supplement: S3 Fig — Each drug response is separated into change in cell death on the Y-axis (as measured by DRAQ7® intensity/spheroid area), and fold change in spheroid count in the X-axis. Each cell line is treated with drugs only (left), and with irradiation (right). Drug classes are shown below the chart. Gy = Gray. (TIF) [file pone.0193694.s003.tif]

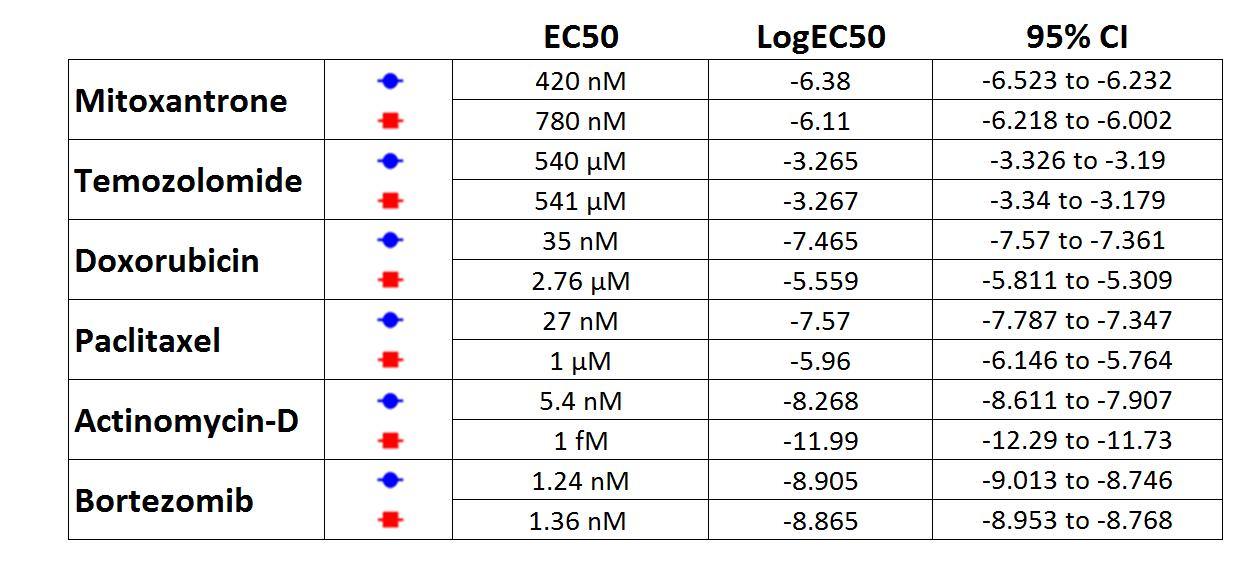

Supplement: S1 Table — The antilog of logEC50s extrapolated from dose response curves were used to populate the table, along with 95% confidence intervals for each EC50. Curves were fitted and EC50 values extrapolated using GraphPad Prism (v7.0). (TIF) [file pone.0193694.s004.tif]
