# Supplementary material for: High content screening of patient-derived cell lines highlights the potential of non-standard chemotherapeutic agents for the treatment of glioblastoma
Source: PLoS One. 2018 Mar 2;13(3):e0193694. doi: 10.1371/journal.pone.0193694 (PMC5834163; doi:10.1371/journal.pone.0193694)
Supplement: S1 File — Additional methods and description of supporting information figures and tables. (DOCX) [file pone.0193694.s005.docx]

# Supplementary Information

# Methods

## Metabolic viability assay

The viability of cells in adherent cultures was evaluated with the resazurin-based PrestoBlue reagent (Life Technologies, UK). PrestoBlue was diluted 1:10 in growth medium and added to each well after aspiration of culture medium. Plates were incubated (37˚C; 5% CO_2_) for thirty minutes as determined from optimisation studies. Fluorescent intensity was detected with a standard plate reader (Synergy™ HT Multi-Detection Microplate Reader, BioTek) at excitation and emission wavelengths of 560 nm and 590 nm, respectively.

## *In vitro* data analysis

Mitoxantrone was used as positive control for analytical purposes after the initial control (staurosporine) failed to produce consistent or meaningful cell death. *In vitro* validation of mitoxantrone was carried out to ensure that the drug produced ≥ 90% cell death in all cell lines at the highest concentration used in the primary screen (data not shown).

For monolayer assays cell death was defined as the percentage of cells with DRAQ7positivity. Defined by setting an upper “circ spot” intensity using the Cellomics compartmental analysis algorithm (Thermofisher, USA). The three dimensional nature of the neurospheres was taken into account by normalizing the total DRAQ7 intensity against spheroid area. Due to variation and non-normal distribution, the values were incremented by 1 and log transformed. Therefore cell death in neurosphere assays was defined as

Log((Draq7 Intensity)/(Spheroid Area)+1)

Cell death data was plotted as a function of the log of each drug concentration and fitted on a variable slope sigmoidal regression model. Log(EC50) was defined as the log of the concentration of drug required to give a cytotoxic response midway between the positive and negative controls.

logEC50=logECF - (1/HillSlope)*log(F/(100-F))

Y=Bottom + (Top-Bottom)/(1+10^((LogEC50-X)*HillSlope))

For monolayer assays, top was constrained at 94 and bottom at 0.7, the mean cell death percentage of mitoxantrone (10 µM) and DMSO vehicle controls respectively. For neurosphere assays the average log cell death of the controls was used to constrain top and bottom, and values were 1.3 and 0.01 respectively. Any drugs that did not give a cell death effect greater than 10% in all assays were removed from the data set. Only log(EC50)s with unambiguous sigmoidal curve fitting were transformed to pEC50 values (the negative logarithm of the EC50) and used to populate heat maps. Agreement between the four HCS assay formats was measured by Bland–Altman limit of agreement plots.

For 96-well plates, the average fluorescent intensity from wells containing treated cells was normalized against that of control wells to determine percent viability of drug-treated cells and EC50 values extrapolated from curves with 95% CI.

pEC50 values were extrapolated from unambiguous dose-response curves generated from DRAQ7 raw data. Dose response curves, heat maps and Bland-Altman plots were generated using Prism (v.7.0, Graphpad Software, USA).

## Response space analysis

For all compounds, the average cell death and spheroid count was calculated across all concentrations. The within concentration variability was calculated and pooled across the concentration levels. All DMSO plate responses were averaged and the associated variability for cell death and spheroid count was determined. Cell death was a composite parameter derived from the total DRAQ7 Intensity per well divided by the Total Spheroid Surface Area, the y-axis represents the difference in cell death between drug and negative control. On the x-axis, as spheroid count was non-normal and log transformed, differences along this axis were expressed as fold change over DMSO as they represented relative rather than absolute differences over DMSO. 95% confidence limits were also calculated for this relative change.

Confidence limits were calculated as follows:

$\bar{X}_{Compound}-\bar{X}_{DMSO}\pm SE\left( diff \right)-t_{df.0975}$

Where

$$SE\left( diff \right)= \sigma- \sqrt{\left[ \frac{1}{n_{Compound}}+ \frac{1}{n_{DMSO}} \right]}$$

Where σ is the pooled standard deviation with the compound concentrations, $t_{df.0975}$ is the 97.5 percentile of values from a Student’s t-distribution with specified degrees of freedom $(df)$. $df$ was defined as $n_{Compound}-5+n_{DMSO}-1$, where $n_{Compound}=20$ and $n_{DMSO}=32$, therefore $df=46$.

A 95% confidence interval means that one can be 95% certain than the true difference between the compound and DMSO value lies within the confidence interval. In the case of cell death, if the confidence interval excludes 0, then there is a statistically significant difference between the test compound and the DMSO value. For spheroid count, if the 95% confidence interval excludes 1, then there is a statistically significant difference between compound and DMSO control.

Statistical analysis was performed using Spotfire (Tibco Software, USA) and data collated and visualised using Microsoft Excel (Microsoft, USA).

# Results

## High content screening

### pEC50 data and drug list

**S1Fig. pEC50 values highlighted general resistance of GBM cell lines and cell line specific responses to chemotherapeutics and irradiation.**

Heat Map was populated using best-fit pEC50 values generated from dose-response curves, then color mapped categorically according to the legend. Each heat map color and corresponding number (1,2,3 etc.), corresponds to a log change in concentration of drug (100,000 µM, 10,000 µM, 1000 µM etc.). Red values of a pEC50 of 9 or above would indicate an EC50 of 1nm or below, indicating high efficacy, whereas any values categorized green-blue or below would likely be poor drug candidates as they correspond to EC50 values above 10µM. Any data Prism was unable to fit was colored black - this was typically due to an inability to generate an EC50 with that drug candidate because of inefficiency at high concentrations.

### Presto Blue Validation Assay

U87 cells were seeded in 96-well plates as standard serum culture to form monolayers, or in serum-free NB media without laminin, to induce neurosphere formation. Plates were run in parallel, with 6 replicates per run of each drug dose and duplicate plates screened 7 days later for additional confirmation. Five of the therapeutics tested were from the primary HCS; mitoxantrone, doxorubicin, paclitaxel, actinomycin-D and bortezomib. Temozolomide was also assayed in both lines to provide a comparison of these drugs efficacy to the current standard of treatment. U87 cells show the similar discrepancies between monolayer and neurosphere culture as seen in the primary HCS.

**S2 Fig. U87 chemosensitivity measured via the Presto Blue assay.**

Dose response curves for five non-standard chemotherapeutics identified in the primary HCS plus the standard GBM therapeutic, temozolomide. Data are presented as the mean cell viability compared to mean vehicle control of two replicate assays (six wells per dose) ± standard error of the mean.

**S1 Table. EC50 values extrapolated from the metabolic viability assay**

The antilog of logEC50s extrapolated from dose response curves were used to populate the table, along with 95% confidence intervals for each EC50. Curves were fitted and EC50 values extrapolated using GraphPad Prism (v7.0).

### Response Space Analysis

**S3 Fig. Neurosphere response space analysis for each cell line.**

Each drug response is separated into change in cell death on the Y-axis (as measured by DRAQ7 intensity/spheroid area), and fold change in spheroid count in the X-axis. Each cell line is treated with drugs only (left), and with irradiation (right). Drug classes are shown below the chart. Gy = Gray.
